# Unilateral gynaecomastia in a 16-month-old boy with neurofibromatosis type 1 – case report and brief review of the literature

**DOI:** 10.3205/iprs000070

**Published:** 2015-12-03

**Authors:** Reinhard E. Friedrich, Christian Hagel, Victor-Felix Mautner

**Affiliations:** 1 Department of Oral and Craniomaxillofacial Surgery, Eppendorf University Hospital, University of Hamburg, Hamburg, Germany; 2 Institute of Neuropathology, Eppendorf University Hospital, University of Hamburg, Hamburg, Germany; 3 Department of Neurology, Eppendorf University Hospital, University of Hamburg, Hamburg, Germany

**Keywords:** neurofibromatosis type 1, gynaecomastia, neurofibroma, breast surgery

## Abstract

Neurofibromatosis type 1 (NF1) is an autosomal dominant disease that shows high penetrance with a wide variability in the phenotype. Prepubertal enlargement of the breast in male subjects affected by this condition is well known, but rarely reported. The present case report describes diagnosis and therapy of unilateral gynaecomastia in a toddler showing integumental stigmata of NF1. Furthermore, the report provides a brief review of the literature concerning this finding in NF1. According to this review, the present case appears to be one of the youngest NF1-affected males affected by gynaecomastia that has been reported.

## Zusammenfassung

Die Neurofibromatose Typ 1 (NF1) ist eine autosomal dominante Erbkrankheit mit hoher Penetranz bei breiter Variabilität des Phänotyps. Präpubertäre Brustvergrößerungen bei männlichen NF1-erkrankten Patienten sind bekannt, aber sehr selten publiziert. Der vorliegende Fallbericht beschreibt Diagnose und Therapie der unilateralen Gynäkomastie eines Kleinkindes mit klinischen Stigmata der NF1 und bietet eine Übersicht bisheriger Fälle mit ähnlichem Erscheinungsbild. Nach Lage der bisher veröffentlichten Berichte handelt es sich in diesem Fall um eine der bisher frühesten Manifestationen einer Gynäkomastie bei NF1.

## Introduction

Gynaecomastia is the development of a breast-like body outline in men. The vast majority of gynaecomastia in children and adolescents are temporary findings, i.e. usually a self-limiting body modification of children and adolescents associated with the onset of puberty. Enlargement of the breasts in small children is rare, especially in males, and deserves medical attention [1]. There are quite a number of malfunctions of the body during early childhood that may be associated with gynaecomastia, in particular endocrinologically active tumours [2], [3]. Nevertheless, the cause of premature breast development is often not discovered. The development of a malignant tumour under the guise of gynaecomastia is predominantly restricted to adults and is a rare event in general [4].

Gynaecomastia is usually bilateral. Unilateral premature breast enlargement is a finding that is difficult to categorise. The rate of pathologies appears to be slightly higher in cases with unilateral gynaecomastia [4]. However, neoplastic tumour development should be considered, in particular in cases with a recognised tumour-predisposition syndrome [5].

Here we present a male toddler with unilateral breast enlargement affected by the tumour-predisposition syndrome neurofibromatosis type 1 (NF1). 

## Report of case

**Figure 1:**
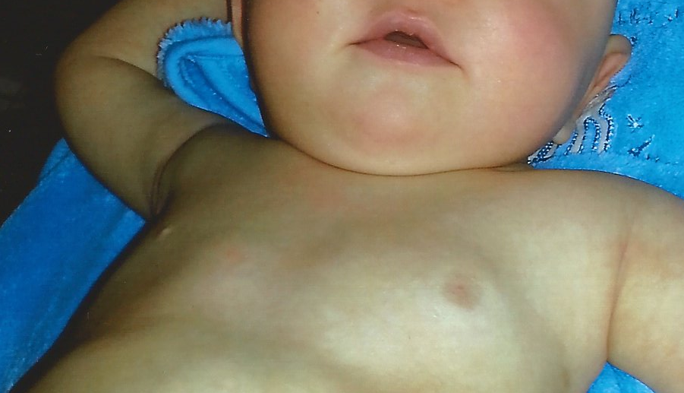
Gynaecomastia in a 16-month-old boy with neurofibromatosis type 1. Note enlarged left breast with inverted nipple.

A 16-month-old boy was presented to the Neurofibromatosis Outpatient Clinic, Eppendorf University Hospital, to evaluate a recently encountered breast finding. About 3 months earlier, the parents had noted a palpable mass below the left areola that slowly increased in size during the next months. On admission, the boy was in excellent general health. The left anterior thorax region showed breast development (Figure 1 (Fig. 1)). The integument was intact and the nipple and areola were of normal size, but the nipple was slightly retracted. The gynaecomastia had developed without any pain and physical investigation of the region was also painless. The pigmentation of the areola and breast showed normal anatomic features and, in particular, there were no colour difference compared to the contralateral region. However, the skin of the affected peri-aureolar region showed a slight pallor (Figure 1 (Fig. 1)). Under digital palpation, the breast felt homogeneously firm and no knots were noticeable. Presumptive diagnosis of the tumour at the time of presentation was neurofibroma.

The patient showed more than six café-au-lait (CAL) spots, which occurred mainly on the trunk, and axillary freckling [6], [7]. The patient showed several xanthogranulomas in the head and neck region. His father was known to be affected by NF1.

## Medical history

**Figure 2:**
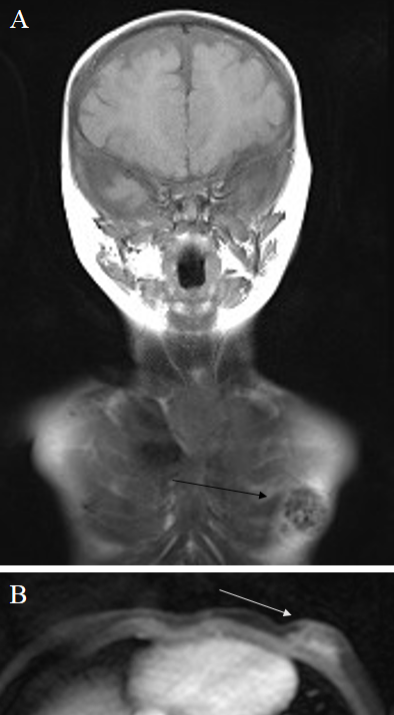
Magnetic resonance images of the NF1-affected child A) On T1-weighted images (coronal plane), an inhomogeneous roundish space-occupying lesion is visible in the left breast region. B) The same region is hyperintense on T2-weighted images (axial plane, cropped image).

The hypertrophic boy was delivered by an unaffected mother 7 weeks prior to the predicted delivery date. Medical records disclosed macrocephaly at birth, in addition to dysregulated muscle tone, neonatal infection and respiratory distress syndrome, and hyperbilirubinaemia. During the next few months, retardation of motor skill development became obvious and CAL-spots of the skin were recorded. Ultrasonographic follow-up investigations of hydrocephalus revealed stable volumetrics of the brain tissues. The ultrasound images of gynaecomastia were repeatedly judged as inflammatory in origin. Prior to surgical therapy, whole-body magnetic resonance imaging (MRI) was performed. MRI showed regular intracranial structures according to age and, in particular, ventricles of normal size and no optic nerve glioma. On the left thoracic side, submammary tissues were identified that were confined to the pectoral soft tissue, weakly hyperintense on T2-weighted images without the typical signal constellation indicating a neurofibroma (Figure 2 (Fig. 2)). Differential diagnosis based on MRI was plexiform neurofibroma or gynaecomastia.

## Therapy

**Figure 3:**
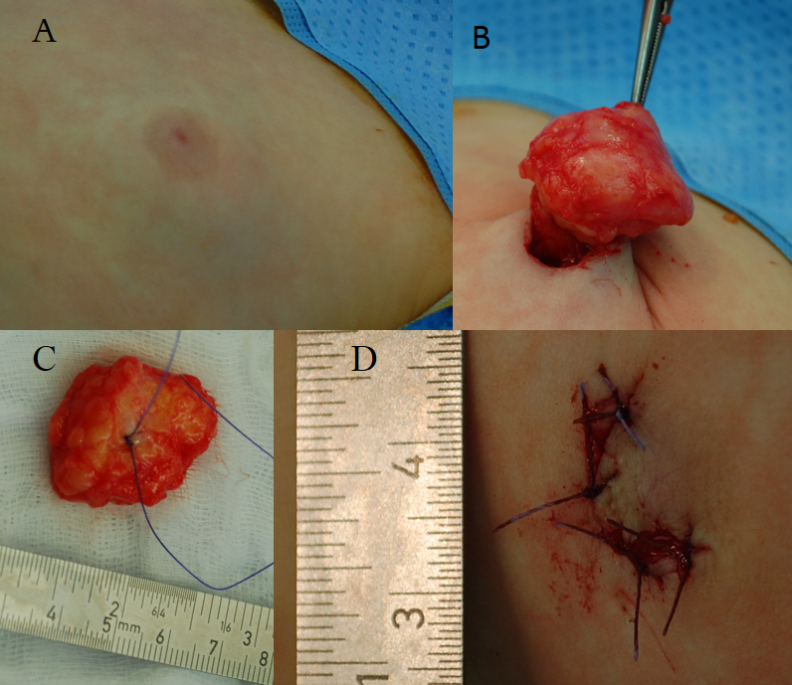
Gynaecomastia in a 16-month-old boy with neurofibromatosis type 1 A) Prominent left breast with peri-areolar pallor. B) Excised tissue after peri-areolar incision and C) tissue with thread tag. D) Closed wound with replaced areola

In order to exclude tumour formation, an operation was carried out by one of the authors in the Department of Oral and Craniomaxillofacial Surgery (REF). A semicircular incision at the medial areola border was chosen to explore the region. Below the areola, the tumour tissue adhered to the retracted skin. The tumor was sharply separated from the skin with a scalpel and completely excised. The tumour looked like fat tissue (Figure 3 (Fig. 3)). Healing was uneventful.

## Histology

**Figure 4:**
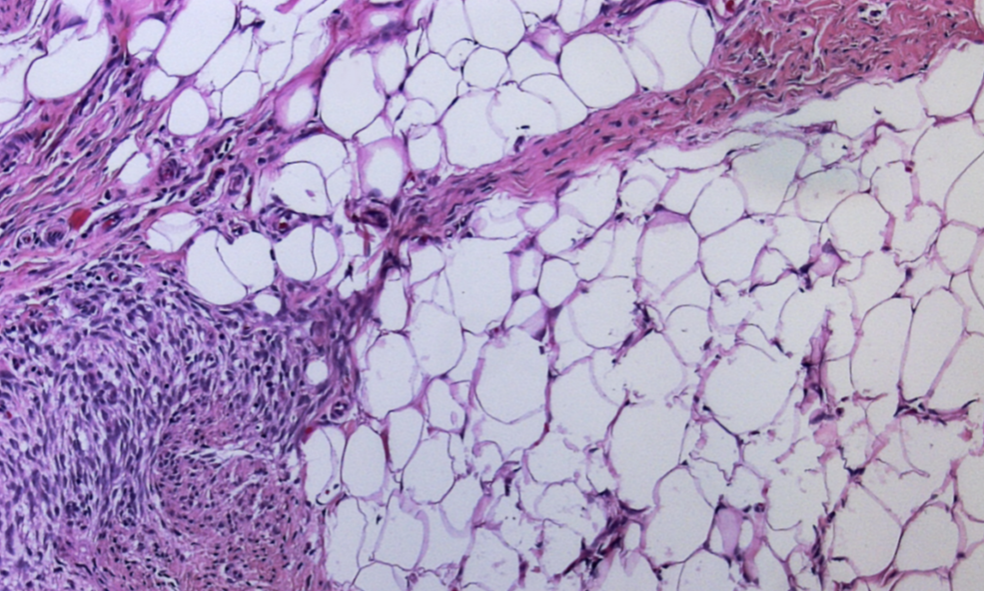
Representative microphotograph of excised breast tissue reveals fat tissue with fibrosis.

The microscopic appearance of the specimen was cellular collagenous connective tissue with isolated glands and multilayered ductal epithelia. The preparation contained some nerve fibre portions but no neurofibroma. Final histological diagnosis was a partially fibrosed mammary gland without any finding indicating neurofibroma. Considering the clinical presentation of the child, diagnosis was gynaecomastia (Figure 4 (Fig. 4)). 

A brief search of the literature on gynaecomastia in NF1-affected children, adolescents and young adults revealed 17 reports on 27 patients (range: 0–25 years) [5], [8], [9], [10], [11], [12], [13], [14], [15], [16], [17], [18], [19], [20], [21], [22]. At least 18 patients were 10 years of age or younger. The mean age of individuals with biographic data (n=24) was 9.1 years at the time of first clinical investigation. The time interval between anamnestically verified first detection of breast swelling and clinical initial examination was on average 2.4 years (n=15). Individual reports are incomplete and some do not indicate the morphological identity of gynaecomastia. In cases where no neurofibroma of the resected enlarged breast was reported (in particular in reports from institutes of pathology), we judged these cases to have no breast neurofibroma. While describing gynaecomastia in neurofibromatosis, some reports were incomplete in terms of an established clinical diagnosis of the underlying disease. Neurofibroma was slightly more frequently associated with (prepubertal) gynaecomastia than increased glandular growth. Individual reports describe only fatty tissue in the resected breast-like tumours. However, terminology of microscopic findings differed considerably. Interestingly, recent reports emphasise the presence of multinucleated stromal giant cells in the tissues. In one case, incidentally bilateral synchronous carcinoma was resected. At least eight patients were of black ethnicity. However, the sample size appears to be too small to draw any conclusion concerning a possible impact of ethnicity on this phenotype. Results are summarised in Table 1 (Tab. 1).

## Discussion

This report details the diagnosis and therapy of unilateral gynaecomastia in a NF1-affected male toddler. This is one of the youngest NF1 patients presenting this alteration in their body outline reported (Table 1 (Tab. 1)). Local excision following a skin incision at the areolar border is sufficient to treat this pathological increase in soft tissue volume. With respect to the presented literature, the histological evaluation of the specimen is mandatory. In NF1-affected male individuals, it is useful to distinguish real gynaecomastia (with glandular enlargement) from pseudogynaecomastia (with neurofibroma ingrowth) [23]. The present case report is about gynaecomastia in NF1. A review of the literature reveals that true gynaecomastia in NF1 is less frequently reported than neurofibroma-associated breast growth in male children (Table 1 (Tab. 1)).

NF1 is a tumour-predisposition syndrome that is characterised by peripheral nerve sheath tumours called neurofibroma. The classification distinguishes cutaneous, diffuse, nodular and plexiform neurofibromas, predominantly based on the synopsis of clinical, radiological and histological findings [24]. In particular, the plexiform neurofibroma (PNF) is classified as a premalignant lesion that gives rise to malignant peripheral nerve sheath tumours (MPNST) [25]. MPNST are very frequently associated with NF1 and the trunk and extremities are the predominant sites of origin, and MPNST may occasionally occur even in children [26]. 

In NF1, early breast development is a well-known finding in females [27]. However, precocious puberty in NF1-affected females is more frequently diagnosed in cases who are suffering from tumours of the hypothalamic/hypophyseal region, in particular optic pathway gliomas [28]. Premature thelarche has to be distinguished from the growth of PNF in the breast region. In the breast region of females, diffuse-invasive and nodular PNF are well-recognised findings that occur in the course of NF1 [29]. These tumours probably develop early in life or are even connately present, but many symptoms may not develop until later in life, e.g. asymmetrical enlargement of the breasts, hyperpigmentation or hirsutism indicating a plexiform neurofibroma under the altered skin. These tumours are usually diagnosed after puberty. Female patients affected by NF1 have a slightly higher risk of developing breast cancer than females in the normal population [30]. Furthermore, specific mutations relevant to the development of hereditary breast cancer have also been reported in NF1 [31]. 

The preferential life stages of males to develop gynaecomastia are the neonatal, pubertal and geriatric ages. The phenomenon occurs due to increased oestrogen exposure of the hormone-dependent mammary glands [32]. Several disease conditions are known to cause this phenomenon, e.g. testicular or gonadal neoplasms, adrenal tumours, oestrogen-containing ointment or drug intake [3]. 

The classic presentation of gynaecomastia is postpubertal in onset and is bilateral, which affects approximately 70% of cases [18]. Precocious gynaecomastia in NF1-affected males is well recognised [8]. However, breast enlargement in male children and adolescents with NF1 is a rarely reported finding [14]. Differential diagnosis of gynaecomastia to plexiform neurofibroma extending into the breast region is mandatory [10]. Occasionally, carcinoma of the breast may arise in males affected by this condition [5]. At present, the data are insufficient to recommend medical therapy in children with idiopathic gynaecomastia [32].

Cho et al. detailed the largest group so far dealing with diagnosis and treatment of gynaecomastia in NF1-affected children and adolescents [18]. These authors reported on six prepubertal, non-obese patients (body mass index <20) with gynaecomastia, who were treated within the age range of 6 to 12 years. Five of six patients were black Americans. Breast enlargement was confined to the nipple-areola complex in four patients, and diffuse involvement was seen in two patients. These authors use the term ‘atypical gynaecomastia’ if the manifestation occurred prepubertally in non-obese children with a preference for unilateral involvement. The patients had extensive endocrinological investigations. However, no abnormal endocrine findings were diagnosed. The authors concluded that endocrine workup is not useful in this group of patients. Treatment was local excision after subareolar skin incision. Healing was uneventful and therapy provided good aesthetic results. Cho et al. [18] differentiated gynaecomastia that was restricted to the areola and diffuse breast involvement. However, the differentiation of gynaecomastia extension should take into account the detection or exclusion of neurofibroma in the resected specimen. Surgical resection is adequate and definitive. No recurrence of gynaecomastia after surgery was seen. However, in one of the published cases, the extension of diffuse breast PNF to a small lumpy mass located just below the areola is visible on photographs [18].

Lubinsky reported on four postpubertal males who had debulking procedures for gynaecomastia that showed fibrous plexiform neurofibroma [27]. We excluded these patients from our study due to the postpubertal occurrence of findings, as well as due to possible overlap of cases in this report to cases included in the Cho et al. [18] report on pseudogynaecomastia in NF1. The latter report explicitly referred to a long-term follow-up of their patients that lasted to the adolescent period and stated that patients with gynaecomastia were shared with Lubinsky [27].

On the other hand, we included the case report of Inglis [33] in our overview, although the use of any diagnostic criteria that are currently mandatory to clinically define NF1 are not apparent from this publication. In this case, neurofibromatosis was considered likely due to the case description and the published images. Interestingly, this author only described adipose tissue in the breast specimen and did not mention neurofibroma in histological sections of amputated digits of the unilateral hyperplastic upper extremity [33].

Some efforts have been made to differentiate the tissue in NF1-associated gynaecomastia, with particular reference to differentiating pseudoangiomatous stromal hyperplasia (PASH) and multinucleated stromal giant cells (MSGC) in NF1-related and non-syndromic gynaecomastia [16], [20], [34], [35], [36], [37]. However, a revaluation of morphological findings in gynaecomastia concluded that these findings are neither specific for the breast alteration nor pathognomonic for NF1 diagnosis [22]. 

## Conclusion

Gynaecomastia is a rare feature in the context of the NF1 phenotype. The lesion is almost always benign in nature. Both gland hypertrophy and neurofibroma can cause breast enlargement, thus giving rise to a physical appearance of breast development in man. The phenomenon is usually not associated with endocrinological malfunction. Surgery is recommended in order to correct an unsightly physical appearance and to clarify the pathogenesis of the lesion. 

## Notes

### Competing interests

The authors declare that they have no competing interests.

## Figures and Tables

**Table 1:**
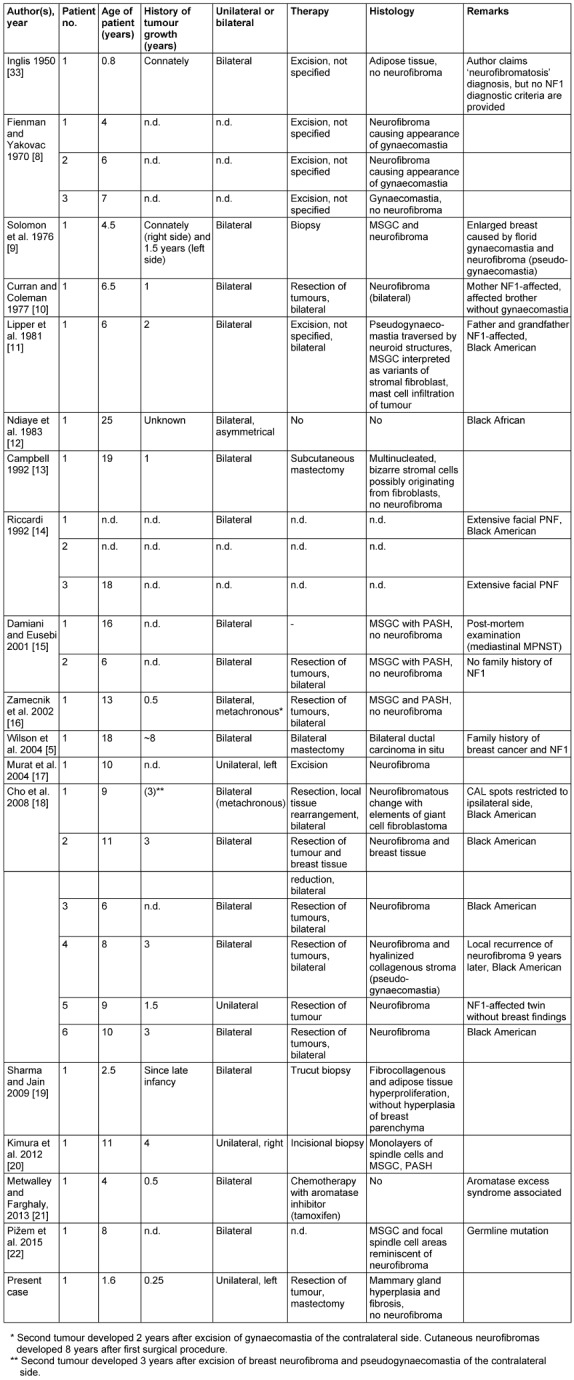
Gynaecomastia in neurofibromatosis (n.d. = not detailed; CAL = café au lait spot; PNF = plexiform neurofibroma; MSGC = multinucleated stromal giant cells; PASH = pseudoangiomatous stromal hyperplasia; MPNST = malignant peripheral nerve sheath tumour)

## References

[1] Yen PP, Sinha N, Barnes PJ, Butt R, Iles S (2015). Benign and Malignant Male Breast Diseases: Radiologic and Pathologic Correlation. Can Assoc Radiol J.

[2] Styne DM (1991). Puberty and its disorders in boys. Endocrinol Metab Clin North Am.

[3] Fagerlund A, Lewin R, Rufolo G, Elander A, Santanelli di Pompeo F, Selvaggi G (2015). Gynecomastia: A systematic review. J Plast Surg Hand Surg.

[4] Lapid O, Jolink F, Meijer SL (2015). Pathological findings in gynecomastia: analysis of 5113 breasts. Ann Plast Surg.

[5] Wilson CH, Griffith CD, Shrimankar J, Douglas F (2004). Gynaecomastia, neurofibromatosis and breast cancer. Breast.

[6] (1988). Neurofibromatosis. Conference statement. National Institutes of Health Consensus Development Conference. Arch Neurol.

[7] Gutmann DH, Aylsworth A, Carey JC, Korf B, Marks J, Pyeritz RE, Rubenstein A, Viskochil D (1997). The diagnostic evaluation and multidisciplinary management of neurofibromatosis 1 and neurofibromatosis 2. JAMA.

[8] Fienman NL, Yakovac WC (1970). Neurofibromatosis in childhood. J Pediatr.

[9] Solomon L, Kim YH, Reiner L (1976). Neurofibromatous pseudogynecomastia associated with prepubertal idiopathic gynecomastia. N Y State J Med.

[10] Curran JP, Coleman RO (1977). Neurofibromata of the chest wall simulating prepubertal gynecomastia. Clin Pediatr (Phila).

[11] Lipper S, Willson CF, Copeland KC (1981). Pseudogynecomastia due to neurofibromatosis--a light microscopic and ultrastructural study. Hum Pathol.

[12] Ndiaye B, Ball MD, Strobel M, Arnold J (1983). Neurofibromatose de Recklinghausen avec énorme tumeur royale et gynécomastie. Ann Dermatol Venereol.

[13] Campbell AP (1992). Multinucleated stromal giant cells in adolescent gynaecomastia. J Clin Pathol.

[14] Riccardi VM (Baltimore). Neurofibromatosis: Phenotype, Natural History, and Pathogenesis. 2nd ed.

[15] Damiani S, Eusebi V (2001). Gynecomastia in type-1 neurofibromatosis with features of pseudoangiomatous stromal hyperplasia with giant cells. Report of two cases. Virchows Arch.

[16] Zamecnik M, Michal M, Gogora M, Mukensnabl P, Dobias V, Vano M (2002). Gynecomastia with pseudoangiomatous stromal hyperplasia and multinucleated giant cells. Association with neurofibromatosis type 1. Virchows Arch.

[17] Murat A, Kansiz F, Kabakus N, Kazez A, Ozercan R (2004). Neurofibroma of the breast in a boy with neurofibromatosis type 1. Clin Imaging.

[18] Cho YR, Jones S, Gosain AK (2008). Neurofibromatosis: a cause of prepubertal gynecomastia. Plast Reconstr Surg.

[19] Sharma R, Jain V (2009). Bilateral breast enlargement in a male toddler: an unusual cause. Indian J Pediatr.

[20] Kimura S, Tanimoto A, Shimajiri S, Sasaguri T, Yamada S, Wang KY, Guo X, Sasaguri Y (2012). Unilateral gynecomastia and pseudoangiomatous stromal hyperplasia in neurofibromatosis: case report and review of the literature. Pathol Res Pract.

[21] Metwalley KA, Farghaly HS (2013). Aromatase excess syndrome presenting with prepubertal gynecomastia in an Egyptian child with type 1 neurofibromatosis. Indian J Hum Genet.

[22] Pižem J, Velikonja M, Matjašič A, Jerše M, Glavač D (2015). Pseudoangiomatous stromal hyperplasia with multinucleated stromal giant cells is neither exceptional in gynecomastia nor characteristic of neurofibromatosis type 1. Virchows Arch.

[23] Lytras A, Tolis G (2009). Reproductive disturbances in multiple neuroendocrine tumor syndromes. Endocr Relat Cancer.

[24] Mautner VF, Hartmann M, Kluwe L, Friedrich RE, Fünsterer C (2006). MRI growth patterns of plexiform neurofibromas in patients with neurofibromatosis type 1. Neuroradiology.

[25] Lévy P, Vidaud D, Leroy K, Laurendeau I, Wechsler J, Bolasco G, Parfait B, Wolkenstein P, Vidaud M, Bièche I (2004). Molecular profiling of malignant peripheral nerve sheath tumors associated with neurofibromatosis type 1, based on large-scale real-time RT-PCR. Mol Cancer.

[26] Friedrich RE, Hartmann M, Mautner VF (2007). Malignant peripheral nerve sheath tumors (MPNST) in NF1-affected children. Anticancer Res.

[27] Lubinsky MS (2006). Non-random associations and vascular fields in neurofibromatosis 1: a pathogenetic hypothesis. Am J Med Genet A.

[28] Listernick R, Ferner RE, Liu GT, Gutmann DH (2007). Optic pathway gliomas in neurofibromatosis-1: controversies and recommendations. Ann Neurol.

[29] Sherman JE, Smith JW (1981). Neurofibromas of the breast and nipple-areolar area. Ann Plast Surg.

[30] Sharif S, Moran A, Huson SM, Iddenden R, Shenton A, Howard E, Evans DG (2007). Women with neurofibromatosis 1 are at a moderately increased risk of developing breast cancer and should be considered for early screening. J Med Genet.

[31] Jeon YW, Kim RM, Lim ST, Choi HJ, Suh YJ (2015). Early-onset breast cancer in a family with neurofibromatosis type 1 associated with a germline mutation in BRCA1. J Breast Cancer.

[32] Ma NS, Geffner ME (2008). Gynecomastia in prepubertal and pubertal men. Curr Opin Pediatr.

[33] Inglis K (1950). Local gigantism (a manifestation of neurofibromatosis): its relation to general gigantism and to acromegaly; illustrating the influence of intrinsic factors in disease when development of the body is abnormal. Am J Pathol.

[34] Badve S, Sloane JP (1995). Pseudoangiomatous hyperplasia of male breast. Histopathology.

[35] Magro G, Amico P, Vecchio GM, Caltabiano R, Castaing M, Kacerovska D, Kazakov DV, Michal M (2010). Multinucleated floret-like giant cells in sporadic and NF1-associated neurofibromas: a clinicopathologic study of 94 cases. Virchows Arch.

[36] Swick BL (2008). Floret-like multinucleated giant cells in a neurofibromatosis type 1-associated neurofibroma. Am J Dermatopathol.

[37] Shaktawat SS, Golka D (2007). Floret-like multinucleated giant cells in neurofibroma. Diagn Pathol.

